# *Fusarium ramigenum*, a novel human opportunist in a patient with common variable immunodeficiency and cellular immune defects: case report

**DOI:** 10.1186/s12879-016-1382-9

**Published:** 2016-02-15

**Authors:** Ruxandra V. Moroti, Valeriu Gheorghita, Abdullah M. S. Al-Hatmi, G. Sybren de Hoog, Jacques F. Meis, Mihai G. Netea

**Affiliations:** National Institute for Infectious Diseases “Prof.Dr.Matei Bals”, Bucharest, Romania; Carol Davila, University of Medicine and Pharmacology, Bucharest, Romania; Carol Davila, Central Emergency University, Military Hospital, Bucharest, Romania; CBS-KNAW Fungal Biodiversity Centre, Utrecht, The Netherlands; Institute of Biodiversity and Ecosystem Dynamics, University of Amsterdam, Amsterdam, The Netherlands; Directorate General of Health Services, Ibri Hospital, Ministry of Health, Muscat, Oman; Department of Medical Microbiology and Infectious Diseases, Canisius Wilhelmina Hospital, Nijmegen, The Netherlands; Department of Medical Microbiology, Radboud University Medical Center, Nijmegen, The Netherlands; Department of Internal Medicine, Center for Infectious Diseases, Radboud University Medical Center, Nijmegen, The Netherlands

**Keywords:** *Fusarium ramigenum* infection, Immune deficiency, Gamma-interferon, IL-17 deficiency

## Abstract

**Background:**

*Fusarium* species are ubiquitous environmental fungi that occasionally provoke serious invasive infections in immunocompromised hosts. Among *Fusarium* species, *Fusarium ramigenum*, belonging to the *Fusarium fujikuroi* species complex, has thus far never been found to cause human infections. Here we describe the first case of invasive fusariosis caused by *Fusarium ramigenum* in a human and also identify immunological deficiencies that most likely contributed to invasiveness.

**Case presentation:**

A 32-year-old Caucasian male with a seemingly insignificant medical history of mild respiratory illness during the preceding two years, developed invasive pulmonary fusariosis. Detailed immunological assessment revealed the presence of common variable immunodeficiency, complicated by a severe impairment of the capacity of T-cells to produce both gamma-interferon and interleukin-17. In-depth microbiological assessment identified the novel human opportunistic pathogen *Fusarium ramigenum* as cause of the infection.

**Conclusion:**

This report demonstrated that an opportunistic invasive fungal infection may indicate an underlying cellular immune impairment of the host. The unexpected invasive infection with *Fusarium ramigenum* in this case unmasked a complex combined humoral and cellular immunological deficiency.

## Background

*Fusarium* species are common saprophytic fungi that globally represent the third cause of invasive mould infection in humans, after *Aspergillus* and after Mucorales. This opportunistic infection is common in Brazil but rare in other parts of the world. The important *Fusarium* species implicated in human pathology belong to the *F. solani*, *F. oxysporum*, and *F. fujikuroi* species complexes [1]. In immunocompetent hosts, clinical manifestations are relatively mild and mostly result from accidental trauma (e.g. keratitis and contact lens-related infections, onychomycosis, osteo-arthritis, but also peritonitis after peritoneal dialysis). Invasive infections are almost exclusively found in immunodeficient hosts, particularly those with severe dysfunction of cellular immunity [2, 3]. In those patients, infections of the respiratory tract are commonly encountered. Mortality due to invasive fusariosis can be above 50 %, even when appropriate and intensive therapeutic management is applied [1, 4]. Here we describe a case of invasive fusariosis caused by a hitherto unknown opportunist, *Fusarium ramigenum*, and report on the immunological causes most likely contributing to this infection.

## Case report

**Fig. 1:**
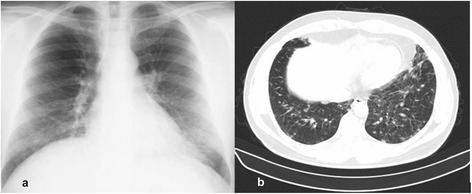
**a** Chest X-ray: bilateral micro-nodular alveolar infiltrates, predominantly in inferior areas; **b** Chest CT-scan: same aspects

A 32-year-old Caucasian male, an outdoor worker (border guard), with mild, recurrent respiratory infections during two preceding years was admitted to the infectious diseases clinic with a 5-day history of high fever, chills, chest pain, dry cough and myalgia. Physical examination showed a good general condition, 38.5 °C fever, no crackles on auscultation, and a palpable spleen (15 cm length). Pulmonary imaging (chest X-ray and lung CT-scan) demonstrated bilateral pulmonary micro-nodular infiltrations and satellite mediastinal lymphadenopathies with a maximum diameter of 16 mm (Fig. 1). Laboratory investigations revealed leukocytosis (15000/mm^3^) with neutrophilia (11700/mm^3^), mild thrombocytopenia (120000/mm^3^), and elevated inflammatory markers (CRP 51 mg/L, ESR 17 mm/h, fibrinogen 416 mg/dl). Serological tests for atypical pathogens (*Chlamydia, Mycoplasma, Coxiella, Legionella*) and Quantiferon for tuberculosis were negative. Blood cultures were also negative.

**Fig. 2:**
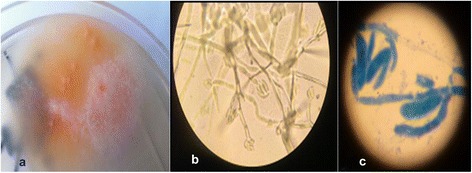
**a** Culture on SGA plate: *Fusarium* colonies; **b** Direct microscopic examination of *Fusarium* with segmented hyphae and conidia x200; **c** Methylene blue stained slide from *Fusarium* culture with banana-shaped conidia, x1200

The initial empirical therapy consisted of moxifloxacin for 2 weeks and non-steroidal anti-inflammatory drugs. The clinical course was unsatisfactory except for a partial decline of fever in the first days but a persistent low-grade fever remained. A broncho-alveolar lavage (BAL) was performed 10 days after admission. Cytology of the BAL fluid was consistent with hemorrhagic lymphocytic alveolitis. No microorganisms were observed during direct microscopical examination. However on the Sabouraud’s glucose agar (SGA) there was a growth of colonies with cottony aerial hyphae which were white, with a light shade of purple and which grew from a pinkish submerged mycelium. The colonies were phenotypically identified as *Fusarium* spp. on the basis of curved, septate conidia (Fig. 2). At this point, invasive fungal infection was not demonstrated and the positive *Fusarium* culture was interpreted as fungal colonization in an apparently immunocompetent patient. Subsequent examination of the patient’s immune system showed a severe hypogammaglobulinemia (0.13 g/l) involving all three analyzed lines: IgM < 0.17, IgG < 0.89, and IgA < 0.24 (g/l). CD4 T-cells were moderately decreased to 468 per cubic mm (33 %), while CD8 T-cells were 745 per cubic mm (53 %), with a low CD4/CD8 ratio (0.63).

Investigations regarding a possible acquired hypogammaglobulinemia (autoimmune diseases, viral infections including HIV, hematologic malignancies) failed to give a clue, suggesting the final clinical diagnosis as being common variable immunodeficiency (CVID). Bone marrow biopsy was normal. The patient was substituted intravenously with immunoglobulins (25 g/day, 5 days). The diagnosis of the patient’s immune deficiency changed the medical judgment of the case, and now an invasive fungal disease being taken into account. Subsequently, voriconazole was added to the therapeutic plan at day 14 after admission (6 mg/kg IV q12h for first 24 h, then 4 mg/kg IV q12h for 2 weeks, then 200 mg orally q12h, with a total duration of six weeks). A voriconazole E test showed an MIC of 2 mg/L. The patient responded with an initial good clinical improvement.

Three weeks after cessation of voriconazole, the patient was re-admitted with productive cough, without fever. Physical examination revealed bilateral, rough vesicular murmurs and a CT-scan showed progressive pulmonary lesions. A significant increase of alveolar infiltrates with extension to the superior regions of the lungs and multiple new spherical dense masses (<5 mm diameter) were observed. A new BAL was performed and the cytology showed the same aspect as few weeks previously (hemorrhagic alveolitis), while the culture was again positive for a *Fusarium* species. IgA, IgG and IgM had again very low values and needed substitution. A second antifungal treatment course with voriconazole was started (same protocol as first course).

**Fig. 3:**
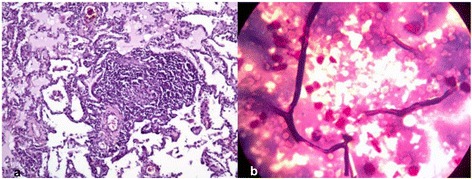
**a** Inflammatory lymphocytic nodular and focal infiltrate with fibrosis (HE stain × 100); **b** Inflammatory reaction and hyphae on pulmonary biopsy smear (Gram stain × 1200)

A lung biopsy was performed at day 8 after voriconazole reinitiation (3 months after first admission). Immunohistochemical examination excluded lung lymphoma and confirmed a reactive cell pattern (interstitial lymphoid infiltrate). Hyaline hyphae were detected in smears from lung tissue imprints (Fig. 3), suggesting an invasive pulmonary fungal disease.

The patient’s immunological status, i.e. the CVID, is a humoral deficit and even in severe forms invasive fungal diseases are rare. Therefore the cellular immune profile was further analyzed and an important qualitative cellular deficiency was additionally found: a defective production of both gamma-interferon- γ (IFN-γ) and IL-17. Deficient cytokine production was demonstrated using a method of whole blood stimulation with specific antigens [5]. The patient’s whole blood IFN-γ production, 72 h after stimulation with heat-killed *Candida albicans* yeast cells (CA), phytohemagglutinin (PHA) and staphylococcal antigen (SA), was 16, 1000 and 12 pg/ml, respectively, and was much lower than the production of 7160, >10000 and 1620 pg/ml of healthy volunteers. IL-17 production after stimulation with PHA was 465 and 300 pg/ml in the volunteers, while values were below detection limit in our patient in both in-duplo stimulations. *Candida albicans* and *S. aureus* did not stimulate IL-17 production in the whole blood stimulation system (Table 1).

**Table 1:** Cytokine production after whole blood stimulation at 24 h (for TNF and for IL-6) and at 72 h (for IFNγ and IL-17)

24 h	IFNγ	Contr.1	Contr.2	Pat.1	Pat.2
RPMI		<78	<78	<78	<78
CA		3270	3130	1120	890
PHA		960	1360	170	220
SA		4810	7480	2430	2560
24 h	IL 6	contr.1	contr.2	pat.1	pat.2
RPMI		32	63	48	26
CA		4700	4100	1730	1560
PHA		1535	1900	265	325
SA		12500	14000	9500	8700
72 h	IFNγ	contr.1	contr.2	pat.1	pat.2
RPMI		10	9	9	<8
CA		8940	7160	16	<8
PHA		8300	>10000	1000	834
SA		1280	1620	12	8
72 h	IL 17	contr.1	contr.2	pat.1	pat.2
RPMI		<40	<40	<40	<40
CA		<40	<40	<40	<40
PHA		300	465	<40	<40
SA		<40	<40	<40	<40

Whole blood was stimulated either with RPMI culture medium (unstimulated control), with heat-killed *C. albicans* (CA), phytohemagluttinine (PHA) or heat-killed *S. aureus* (SA). Concentrations of the cytokine produced are expressed as pg/mL

The evolution was favorable under prolonged antifungal therapy with voriconazole for 6 months and continuous immunoglobulin substitution with 25 g/day, 5 days per month. A CT-scan after 6 months showed regression of the pulmonary lesions. The subsequent BAL was culture-negative for *Fusarium* and no signs of hemorrhagic lymphocytic alveolitis were seen. Antifungal treatment was stopped and during two years of follow-up (CT-scan, respiratory functional tests) no further progression was noted.

**Fig. 4:**
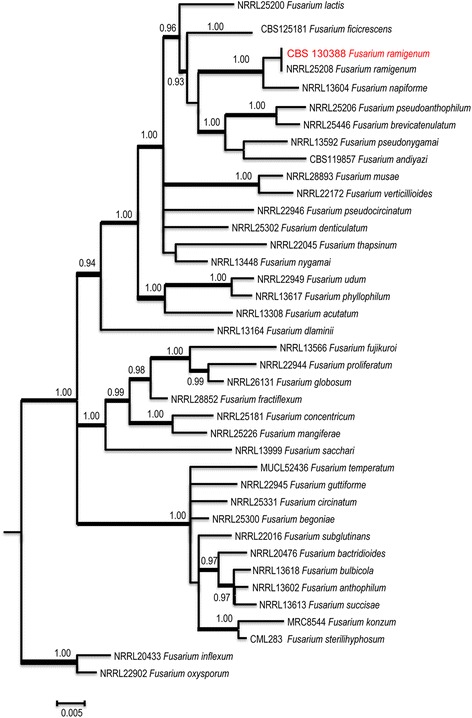
Phylogenetic analysis of *Fusarium ramigenum.* Phylogenetic reconstruction obtained from Bayesian inference of two combined loci (*TEF1* and *BT2*) using MrBayes v. 3.1.2. Values at branch node indicate branch support with posterior probabilities (PP; values ≥ 0.80 shown) and branches in boldface = bootstrapping percentages based on Maximum Likelihood (ML). The tree was rooted with two strains of *F. inflexum* NRRL20433 and *F. oxysporum* NRRL22902

Further identification of the fungus was undertaken at the CBS-KNAW Fungal Biodiversity Centre in Utrecht, The Netherlands, under accession number CBS 140388. Sequencing of partial elongation factor 1 alpha (*TEF1*) and beta-tubulin (*BT2*) genes was performed. Blast results with sequences in GenBank revealed that this fungus belonged to the *Fusarium fujikuroi* complex. In order to establish the phylogenetic position of this clinical isolate, a general tree was made with MrBayes v. 3.1.2 on the Cipres Portal based on the sequenced *BT2* (500 bp) and *TEF-1* (600 bp) regions. Thirty-six species within the *Fusarium fujikuroi* species complex were selected for phylogenetic analyses of combined *BT2* and *TEF1* fragments. Our strain was nested with a *F. ramigenum* subclade (Fig. 4). Sequences of this novel human opportunistic fungus (CBS 140388) were deposited in GenBank with accession numbers KT794172 for *BT2* and KT794175 for *TEF1*, respectively.

Antifungal susceptibility testing performed with broth microdilution according to CLSI M38A resulted in the following MICs/MECs: amphotericin B, 1 mg/L; posaconazole, 1 mg/L; itraconazole, >16 mg/L; voriconazole, 2 mg/L; isavuconazole, 4 mg/L and anidulafungin and micafungin both > 8 mg/L.

## Discussion

We describe a patient with CVID and cellular T-helper-defects who developed an invasive infection with *Fusarium ramigenum*. After long-term treatment with voriconazole (6 months) and immunoglobulin substitution, the patient recovered from this opportunistic infection. To the best of our knowledge, this is the first case in which *F. ramigenum* was described as a cause of invasive infection in a human patient, reinforcing the significance of the *F. fujikuroi* species complex as opportunistic pathogens in immunocompromised hosts. The fungus cultured from BAL specimens of this patient with atypical pneumonia and no significant medical history first suggested fungal colonization rather than infection. Moreover, the observed BAL cytology, indicating hemorrhagic lymphocytic alveolitis, could neither prove nor exclude a fungus induced inflammatory reaction. However, the relapse of *Fusarium* infection after a short 6-week course of antimycotic therapy (6 weeks) raised the suspicion of an invasive infection. The microscopic findings of hyaline hyphae in the lung biopsy confirmed the invasive fusariosis. This was further supported by the identification of combined humoral (severe pan-hypogammaglobulinemia) and cellular (defective IFN- γ and IL-17 production capacity) immune defects that are known to be crucial for antifungal host defense [6, 7].

The initial humoral immunological deficit identified, i.e. severe pan-hypogammaglobulinemia, was not consistent with an invasive fungal infection. The slightly lowered CD4 T-cell counts, combined with a reduced T-cell CD4:CD8 ratio, could not explain this opportunistic infection either, and therefore we embarked on functional assays to test the T-helper functions. Subsequent analysis revealed an important deficiency, with very low levels of IFN-γ and a deficit in IL-17 production capacity. Cellular defects in CVID patients have been reported previously, and this is the most likely explanation of the observed infection [8, 9]. The results are in consensus with the latest data in the literature, describing the possibility of complex T-cell abnormalities in association with CVID. T-cell abnormalities associated with CVID generate a slight quantitative deficit of CD4 lymphocytes, an abnormal CD4/CD8 ratio, and a qualitative deficit in cytokine production [10–14]. The exact mechanisms and genetic causes of these defects in CVID remain to be elucidated. Alternatively, a different explanation may be represented by defects in genes known to be crucial for antifungal host defense, such as the CARD9 adaptor [6, 7].

An important aspect of this clinical case is the first identification of a novel human opportunistic fungus, *F. ramigenum* as cause of the infection. This fungus belongs to the relatively frequently encountered *F. fujikuroi* complex, but molecular analysis identified *F. ramigenum*, a species not figuring on the list of species known to occur in human or animal infections [15]. *Fusarium ramigenum* was first described in 1998 from inedible wild Capri figs in California, U.S.A. [16]. The species produced fusaric acid, beauvericin and fumonisin [17]. Its pattern of susceptibility to antimycotic therapy showed potential activity of amphotericin B, voriconazole and posaconazole and no activity of itraconazole and the echinocandins which is similar to a previous study [18]. The relevance of these in vitro data is not clear because a correlation between MICs/MECs and clinical outcome has not been documented for fusariosis [2]. The MIC of voriconazole of 2 mg/L is below the mode of 4 mg/L as described for *F. fujikuroi* [19] and a retrospective analysis of 73 patients with invasive fusariosis showed a 47 % success rate [20]. Indeed recently published guidelines recommend voriconazole (AII) or amphotericin B (BII) as treatment option for invasive fusariosis [21].

## Conclusion

In summary, this report demonstrated that an opportunistic invasive fungal infection may indicate an underlying cellular immune impairment of the host. The unexpected invasive infection with *F. ramigenum* in this case unmasked complex combined humoral and cellular immunological deficiencies. Moreover, this paper provides evidence indicating *F. ramigenum* as a potential human opportunist especially in immunocompromised patients.

## Consent

Written informed consent was obtained from the patient for publication of this case report and any accompanying images. A copy of the written consent is available for review by the Editor of this journal.

## Availability of supporting data

The phylogenetic tree supporting the results of this article is available in the [TreeBASE] repository, [http://purl.org/phylo/treebase/phylows/study/TB2:S18586?x-access-code=b4893a30c99ca55b93a029cbdae331a8&format=html].

## Abbreviations

CT-scan: Computed tomography
CRP: C-reactive protein
ESR: Erythrocyte sedimentation rate
BAL: Broncho-alveolar lavage
SGA: Sabouraud’s glucose agar
IgM: Immunoglobulin M
IgG: Immunoglobulin G
IgA: Immunoglobulin A
CD4: Cluster of differentiation 4
CD8: Cluster of differentiation 8
HIV: Human immunodeficiency virus
CVID: Common variable immunodeficiency
CA: Candida albicans yeasts
PHA: Phytohemagglutinin
SA: Staphylococcal antigen
IL-17: Interleukin 17
TEFI1: Translation elongation factor1
BT2: Beta-tubulin
CLSI: Clinical and Laboratory Standards Institute
CLSI M38-A: CLSI microtiter mould testing standard methods for antifungal susceptibility
MIC: Minimum inhibitory concentration
IFN γ: Gamma-interferon
CARD9: Caspase recruitment domain-containing protein 9
